# The safety and efficacy of lung volume reduction surgery: A 25-year single institution experience

**DOI:** 10.1016/j.xjon.2026.101722

**Published:** 2026-04-01

**Authors:** Gian-Marco Monsch, Bianca Battilana, Claudio Caviezel, Dario Gregori, Ester Rosa, Adriana Grabar, Kathrin Chiffi, Carolin Steinack, Didier Schneiter, Isabelle Opitz

**Affiliations:** a Department of Thoracic Surgery, University Hospital Zurich, Zurich, Switzerland; b Department of Thoracic Surgery, Canton Hospital Chur, Chur, Switzerland; c Unit of Biostatistics, Epidemiology and Public Health, University of Padua, Padua, Italy; d Department of Pulmonology, University Hospital Zurich, Zurich, Switzerland; e University of Zurich, Zurich, Switzerland

**Keywords:** lung volume reduction surgery, COPD, emphysema, BMI, hypercapnia

## Abstract

**Objective:**

Lung volume reduction surgery (LVRS) in well-selected patients significantly improves lung function. Regardless of its proven effects, LVRS is still widely underused worldwide. In this overview, we demonstrate our 25 years of experience in the largest single-center cohort.

**Methods:**

Retrospective analysis of prospectively collected data on patients undergoing LVRS at the Department of Thoracic Surgery, University Hospital Zurich between November 2000 and February 2025 is presented, including functional outcomes.

**Results:**

Seven hundred thirteen patients (323 women [45.3%]) with a median age of 65 years (range, 59-71 years) and predominantly heterogeneous morphology (70.3%) needing in 38.9% permanent oxygen supply underwent LVRS (93.7% minimally invasive approach, 43.1% bilateral). All relevant lung function parameters had improved significantly at the 6-month follow-up (change [Δ] forced expiratory volume in 1 second: +22.0% predicted, Δ residual volume: –762.2 mL, Δ total lung capacity: –418.1 mL, Δ residual volume to total lung capacity ratio: −10.3%, Δ diffusing capacity of the lungs for carbon monoxide: +3.2, and Δ 6-minute walk distance: +32 m; all *P* values < .001). The most common postoperative complication was prolonged air leak (27.2%), necessitating redo surgery in 78 (10.9%) patients. In-hospital mortality was 0.7%, and 90-day mortality was 2.1%. The 1- and 5-year survival rates were 93.4% and 52.1%, respectively. Body mass index below 18.5 and higher intraoperative Paco_2_ were associated with poorer survival.

**Conclusions:**

LVRS leads to a significant improvement of all key lung function parameters, with low in-hospital mortality in this very fragile patient cohort.

## Graphical Abstract

**Figure:**
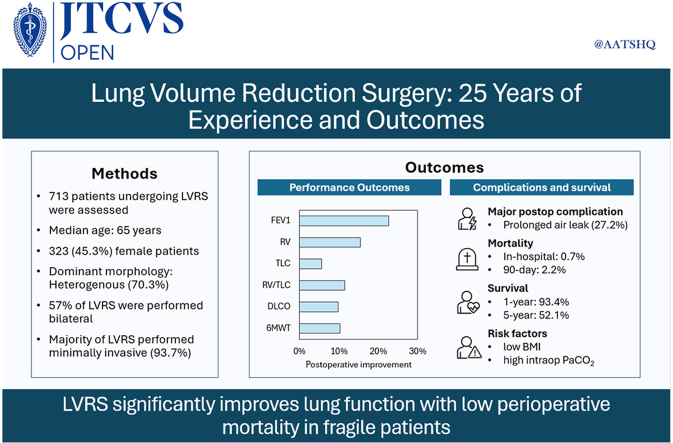

**Figure:**
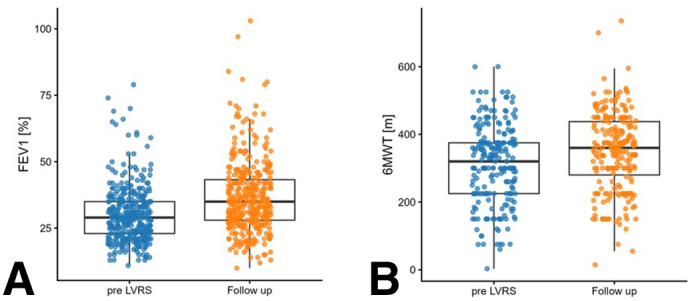
Pre-/postoperative FEV1 (A) and 6MWD (B) in patients undergoing LVRS (∗∗∗*P* < .001).

**Central Message**

LVRS significantly improves all key pulmonary function parameters and 6MWD performance. In experienced centers, these benefits can be achieved with an in-hospital mortality rate <1%.

**Perspective**

Optimizing outcomes in patients undergoing LVRS requires meticulous perioperative multidisciplinary management. Elevated Paco_2_ at induction of surgery may serve as a valuable parameter for risk stratification if independently validated. Improving nutritional status during a prehabilitation phase in patients with low BMI could enhance perioperative resilience and optimize functional results.

Chronic obstructive pulmonary disease (COPD) is a globally relevant health problem and the fourth most common cause of death with more than 3 million deaths per year.^1^ Chronic emphysematous destruction of the lungs leads to shortness of breath and a concomitant decline in physical performance and quality of life. Lung volume reduction surgery (LVRS) has been proven in several prospective and a few randomized, controlled trials to be a successful palliative treatment for selected patients.^2^^,^^3^ Nevertheless, currently, LVRS is worldwide not largely used, or only hesitantly (eg, between 2017 and 2022, only 244 LVRSs were reported in the UK Lung Volume Reduction Registry^4^). In particular, patients with homogeneous emphysema are not even evaluated for LVRS in many centers, potentially stemming from a subgroup analysis of the National Emphysema Treatment Trial (NETT) from 2001, which describes high mortality after LVRS in patients with homogeneous emphysema. This misinterpretation persists stubbornly, although the credo has been disproven in numerous publications.^5^^,^^6^

To help dispel the long-standing negative perception of LVRS, we present to the best of our knowledge the largest single-center cohort to date, backed by more than 25 years of expertise, detailed functional outcomes, and advanced modeling over the period. Institutional review board approval was provided: BASEC 2020-02566 (09.02.2021). Data were collected, analyzed, and published based on written informed consent to the “RIO” project.

## Patients and Methods

### Study Design

This is a retrospective, monocentric cohort study from November 2000 until February 2025, which included 713 patients undergoing LVRS in our department. Patient data were prospectively collected and extracted from the electronic medical record. The study was carried out in accordance with principles set out in the current version of the Declaration of Helsinki and the guidelines of Good Clinical Practice. The project was approved by the Zurich Ethics Committee (BASEC No. 2020-02566, 09.02.2021).

### Patient Selection and Management

**Figure 1:**
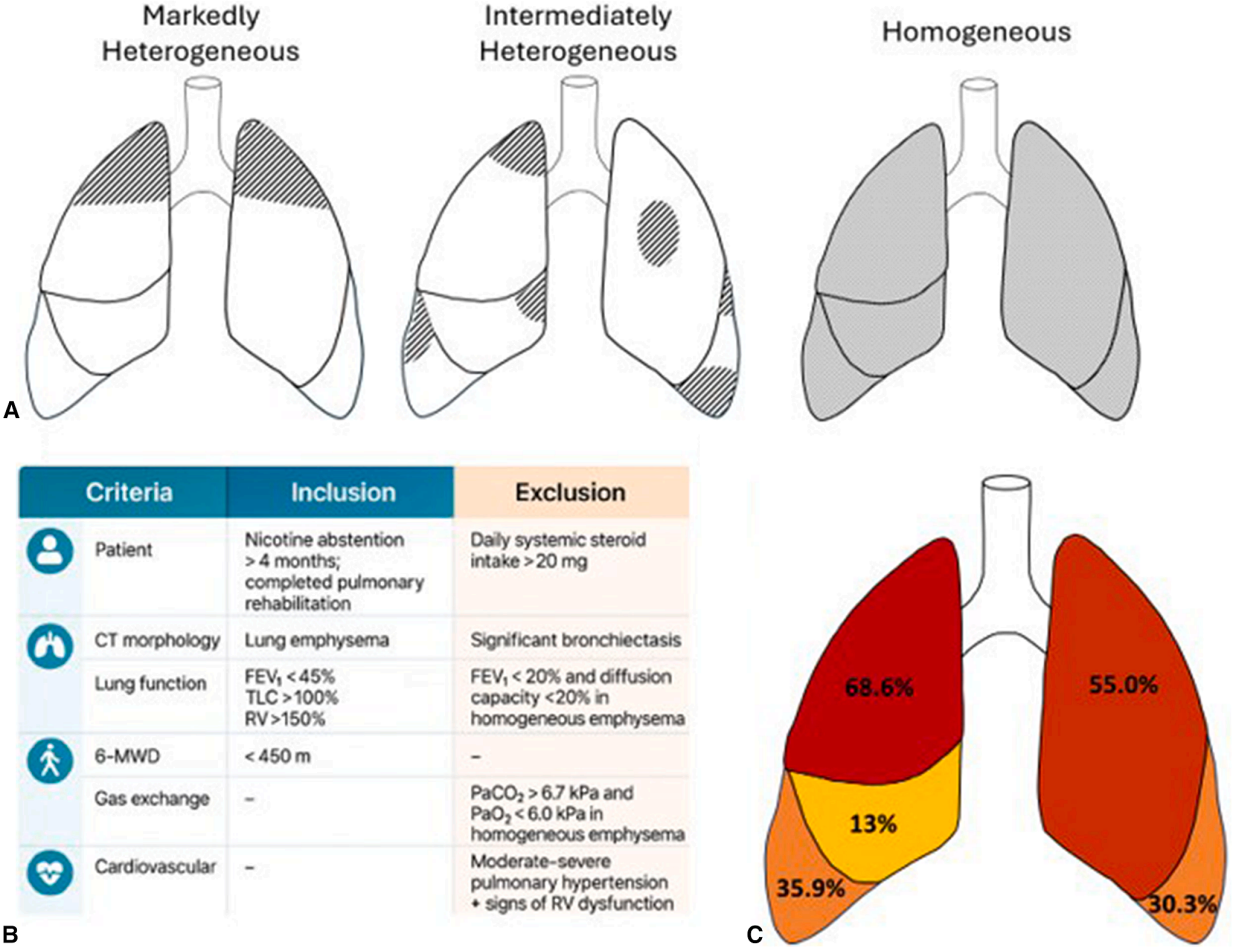
A, Overview of emphysema morphology. B, Selection criteria for lung version reduction surgery (*LVRS*). C, Target zone for LVRS, percentage indicates how often the lobe was targeted out of all conducted surgeries. Because often more than 1 lobe is targeted, the percentage does not add up to 100%. *CT*, Computed tomography; *FEV1*, forced expiratory volume in 1 second; *RV/TLC*, residual volume to total lung capacity ratio; *6-MWD*, 6-minute walk distance; *RV*, right ventricle.

Every potential LVRS candidate underwent a standardized preoperative functional assessment: exhausted conservative treatment options, body plethysmography, transthoracic echocardiography, and right heart catheterization if necessary (estimated systolic pulmonary artery pressure >35 mm Hg) as well as dual energy computed tomography scan and lung perfusion scintigraphy. For optimal treatment allocation (LVRS vs endobronchial valves vs direct lung transplant candidacy), every patient is discussed in an interdisciplinary emphysema board consisting of thoracic surgery and pneumology. Emphysema morphology was classified corresponding to the categorization published in 1997^7^ (Figure 1, *A*), based on computed tomography scan and perfusion imaging and agreed upon in the interdisciplinary emphysema board. Selection criteria for surgery are summarized in Figure 1, *B*.^8^ Over time, there were 2 main refinements of our selection criteria: firstly, prioritizing residual volume to total lung capacity ratio (RV/TLC) over RV and TLC values because it’s a more clinically relevant indicator of static hyperinflation, remaining valid even in homogenous disease^9^; secondly, inclusion of patients with homogeneous subtype of emphysema with clear hyperinflation (RV/TLC >0.6) and absence of risk factors (severe pulmonary hypertension or diffusing capacity of the lungs for carbon monoxide [DLCO] <20%) as potential candidates for surgery. In bilateral emphysema distribution, we aim for bilateral LVRS. In clearly unilateral distribution or previous surgeries, unilateral LVRS is considered.

Perioperative management is highly standardized: 3-portal video-assisted thoracoscopic surgery; adhesiolysis without the use of electronic devices; resection with reinforced stapler maintaining the lung configuration; depending on resected volume and location, release of pulmonary ligament; or peridural anesthesia for bilateral LVRS or intercostal blocks in unilateral cases. In all cases, a double-lumen tube is switched to a laryngeal mask before extubating to prevent coughing. Postoperatively, patients are transferred to the intermediate care unit. All patients are mobilized on the day of surgery. Antibiotic prophylaxis is given until removal of the last chest drain (usually 1 drain per side), which occurs after an evaluation of a leak at the Heimlich valve and bag.

### Statistical Analysis

Descriptive statistics summarize continuous variables as medians and interquartile ranges (IQR), and categorical variables as counts and percentages. Follow-up assessments at baseline, 3, 6, 9, and 12 months were considered, in accordance with the time horizon commonly adopted in the literature. Because not all patients had data at every time point, the first and last available measurements within this 12 months were used.^10^

Functional outcome analyses included patients with both a pretreatment measurement and at least 1 follow-up within 3 to 12 months (with 6 months standard; 3, 9, or 12 months accepted if 6-month assessment was unavailable). Using a single harmonized cohort ensured comparability across outcomes. Extreme within-subject changes were excluded using a conservative 3 × IQR rule, and pre-post differences were evaluated using Wilcoxon signed-rank tests. Availability of complete pre-post data is reported in Table E1.

Imputation of missing values was not performed because the analyses relied on within-patient pre-post comparisons. Only patients with at least 2 valid measurements (baseline and 1 postoperative value within the predefined follow-up window) contribute to the estimation of functional changes; therefore, imputing values would require additional model-based assumptions and would not provide independent empirical information. To avoid introducing potentially unverifiable assumptions about postoperative trajectories, a transparent complete-case approach for all pre-post comparisons was adopted.

### Survival Analysis

Postoperative survival was estimated using the Kaplan-Meier method. Overall survival was calculated from the time of LVRS to death or last follow-up, irrespective of subsequent lung transplantation, with 95% CI. Effects were also interpreted on the IQR. Group comparisons were performed using log-rank tests. Additional analyses assessed survival differences across preoperative body mass index (BMI) categories and emphysema morphology. Patients were classified into 3 BMI categories: underweight (<18.5), normal (18.5 to 25), and overweight/obese (>25). Survival across surgical periods was evaluated using Kaplan-Meier analyses, grouping years into 5-year intervals.

Associations between preoperative variables and survival were evaluated by multivariable analyses using Cox proportional hazards models. Nonlinear effects of BMI, Paco_2_ at induction of surgery, and cohort were modeled with restricted cubic splines, and functional form was assessed via spline-based log-hazard ratio plots with 95% CI. To account for the potential cohort effect in the calendar time when surgery was performed, a frailty term for the year of surgery was introduced in the model. To account for potential within-cluster correlation and heteroscedasticity in the data, a robust marginal model was used.^11^ Effects of continuous predictors were expressed as regression coefficients multiplied by the IQR for scale-independent comparison.

### Predictors of Lung Function

Predictors of postoperative lung function were evaluated using ordinary least squares regression models applied to repeated measurements of forced expiratory volume in 1 second (FEV1), TLC, RV, and DLCO. Models included restricted cubic splines for time (in months) to capture nonlinear postoperative trends and covariates: age, BMI, Paco_2_ at surgery, COPD Global Initiative for Chronic Obstructive Lung Disease (GOLD) classification, emphysema morphology, presence of LVRS, and postoperative complications. Cluster-robust SEs (Huber-White sandwich estimator) with clustering at the patient level^12^ accounted for within-patient correlation and heteroskedasticity, preserving consistency and marginal interpretation. A multivariable logistic regression was performed to identify predictors of postoperative complications, including prolonged air leak (PAL). Model fit was assessed with likelihood ratio and Wald χ^2^ tests; odds ratios (ORs) were reported with 95% CI. Initial models used splines for BMI and Paco_2_, but final models applied linear terms after confirming no relevant nonlinearity.

Model assumptions were checked using Schoenfeld residuals and visual diagnostics. For all multivariable models, Wald-type analysis of variance tested joint significance of covariate effects, including spline components and categorical contrasts. This approach allowed global hypothesis testing beyond individual parameter estimates, providing a comprehensive evaluation of each variable's contribution to model fit. For all Cox proportional hazards models, internal validity was assessed using bootstrap resampling (10 000 repetitions). Internal validity for Cox models was assessed using 10 000 bootstrap resamples. Discrimination was evaluated with Harrell's discrimination index, converted to concordance indices for interpretation. All analyses were carried out with R Studio (R Foundation for Statistical Computing).^13^

## Results

### Study Population

A total of 713 patients underwent LVRS at our institution between November 2000 and February 2025. Baseline characteristics are shown in Table 1. Baseline characteristics stratified by surgical quinquennium and emphysema morphology are presented in Tables E2 and E3).

**Table 1:** Baseline characteristics of the patients undergoing lung volume reduction surgery

Characteristic	Result
Age (y)	65 (59-71)
Sex	
Male	390 (54.7)
Female	323 (45.3)
COPD classification	
I	2 (0.28)
II	14 (1.96)
III	198 (27.77)
IV	399 (55.96)
Pack-years	45 (40-60)
Side of emphysema	
Unilateral	171 (23.98)
Bilateral	542 (76.02)
Type of emphysema	
Heterogeneous	501 (70.27)
Homogeneous	112 (15.71)
Intermediate	100 (14.03)
Oxygen usage	
Yes	277 (38.85)
No	436 (61.15)

Values are presented as median (interquartile range Q1-Q3) or n (%). *COPD*, Chronic obstructive pulmonary disease.

### Perioperative Outcomes

Perioperative results are summarized in Table 2. The most frequently targeted lobe for LVRS was the right upper lobe (68.6%) (Figure 1, *C*). No major intraoperative complications occurred, except 9 cases (1.3%) of substantial air leakage (>1000 mL/min) that required additional intraoperative measures. The most frequent postoperative complication was PAL (>7 days), observed in 194 patients (27.2%).

**Table 2:** Perioperative outcomes after lung volume reduction surgery (LVRS) (N = 713)

Patients undergoing LVRS	Result
LVRS type	
Bilateral	406 (57)
Unilateral	307 (43)
LVRS target	
Right upper lobe	489 (68.6)
Right middle lobe	93 (13.0)
Right lower lobe	256 (35.9)
Left upper lobe	392 (55.0)
Left lower lobe	216 (30.3)
Hospitalization duration (d)	10.0 (7.0, 14.0)
Chest drainage duration (d)	5.0 (4.0, 10.0)
Postoperative complications	
No	470 (66)
Yes	243 (34)
Type of postoperative complications	
Prolonged air leak	194 (27.2)
Empyema	8 (1.1)
Pneumothorax	4 (0.6)
Hemothorax	10 (1.4)
Prolonged weaning	4 (0.6)
Other complications	21 (2.9)
ICU stay duration (d)	1.00 (0.00, 1.00)
In-hospital mortality	
Alive at discharge	708 (99.3)
Died in hospital	5 (0.7)
90-d mortality	
Dead	15 (2.1)
Patient status	
Alive	158 (22)
Dead	470 (66)
Lost to follow-up	85 (12)

Values are presented as median (interquartile range Q1, Q3) or n (%). *ICU*, Intensive care unit.

Predictors of postoperative complications were assessed using 2 multivariable logistic regression models (Tables E4 and E5). For any complication, COPD GOLD classification and emphysema morphology were significant predictors, whereas BMI, age, and Paco_2_ at induction of surgery were not (Table E4). For PAL, lower BMI (OR, 0.82; 95% CI, 0.73-0.92; *P* = .018), higher Paco_2_ at induction of surgery (OR, 1.20; 95% CI, 1.08-1.34; *P* = .0009), and older age (OR, 1.28; *P* = .006) were independently associated (Table E5). Median hospital stay was 10 days (IQR, 7-14 days), and median chest drainage duration was 5 days (IQR, 4-10 days). Overall, 34% of patients experienced at least 1 postoperative complication, whereas 66% had an uneventful course. In-hospital mortality was 0.7% (n = 5), 90-day mortality was 2.1% (n = 15). One-, 3-, and 5-year survival was 93.8% (range, 91.9%-95.7%), 76.0% (72.5%-79.7%), and 55.1% (50.9%-59.7%), respectively. Median follow-up was 4.3 years (IQR, 2.2-7.2 years). At follow-up end, 22% were alive, 66% had died, and 12% were lost to follow-up.

### Functional Outcomes

Functional outcomes are presented in Table 3. All major pulmonary function parameters and the 6-minute walk distance (6MWD) improved significantly (all Bonferroni-adjusted *P* values < .001). FEV1 increased from 770.5 to 944.9 mL, corresponding to an improvement of 22.0%. Measures of hyperinflation showed substantial reductions, with RV decreasing by 762.2 mL, TLC by 418.2 mL, and RV/TLC by 10.3%. Gas exchange improved modestly but significantly, with DLCO increasing by a mean of 3.2 units. Exercise capacity also improved, with the 6MWD increasing by an average of 32 m. Data completeness varied across functional parameters (Table E1), but improvement direction and magnitude were consistent, confirming robust and clinically meaningful benefits after LVRS.

**Table 3:** Baseline and last available follow-up values for pulmonary function outcomes

Outcome	n	Baseline	Follow-up	Δ	% Δ	*P* value∗
FEV1 (mL)	352	777.9	948.5	170.6	21.9	<.001
FEV1 (%)	360	29.5	36	6.5	22.0	<.001
RV (mL)	365	5174.3	4412.1	−762.2	−14.7	<.001
RV (%)	363	231.1	196.8	−34.3	−14.8	<.001
TLC (mL)	362	7660	7241.9	−418.1	−5.5	<.001
TLC (%)	358	131.2	123.4	−7.8	−5.9	<.001
RV/TLC	365	67.2	60.3	−6.9	−10.3	<.001
DLCO	360	32.9	36.1	3.2	9.7	<.001

Values are presented as mean unless otherwise noted. *Δ*, Change; *FEV1*, forced expiratory volume in 1 second; *RV*, residual volume; *TLC*, total lung capacity; *RV/TLC*, residual volume to total lung capacity ratio; *DLCO*, diffusing capacity of the lungs for carbon monoxide.

∗ *P* values refer to the latter and were adjusted for multiple comparisons using the Bonferroni correction.

### Predictors of Postoperative Functional Improvement

**Figure 2:**
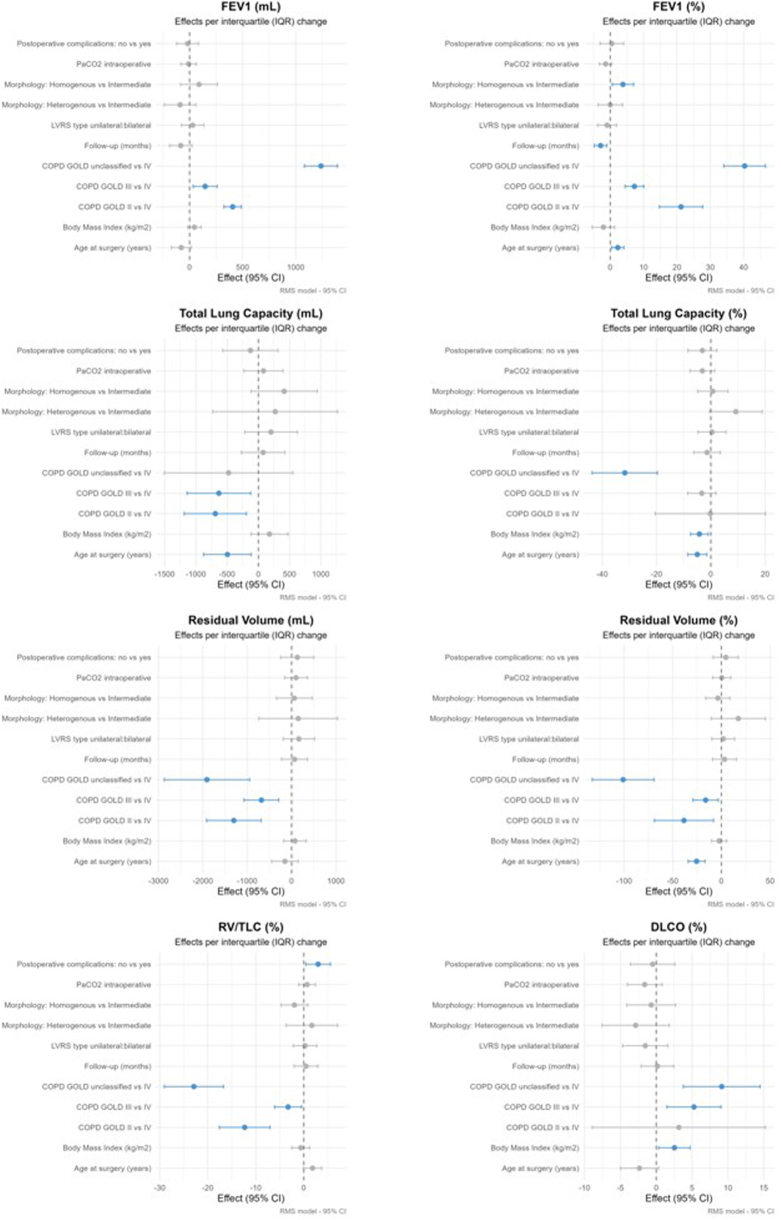
Forest plots of effects estimate for postoperative lung function outcomes after lung version reduction surgery (*LVRS*), with 95% CI. Values in brackets represent quartile I, quartile III. Estimates shown in blue indicate statistically significant effects. *FEV1*, Forced expiratory volume in 1 second; *RV/TLC*, residual volume to total lung capacity ratio; *DLCO*, diffusing capacity of the lungs for carbon monoxide.

A significant effect over time was observed across all outcomes, indicating progressive improvement in lung function after LVRS. Older age was linked with smaller gains, particularly for RV and TLC, whereas higher BMI correlated with better DLCO and lower RV/TLC, suggesting more efficient gas exchange and less residual hyperinflation. Advanced COPD (GOLD classification III or IV) showed limited reduction in hyperinflation, whereas heterogeneous emphysema morphology yielded greater FEV1 improvement. Bilateral LVRS was associated with slightly larger RV and RV/TLC reduction than unilateral surgery. Results are summarized in Table E6. Corresponding forest plots (Figure 2) highlight emphysema morphology, disease severity, and BMI as dominant predictors of postoperative recovery.

### Predictors of Survival

**Figure 3:**
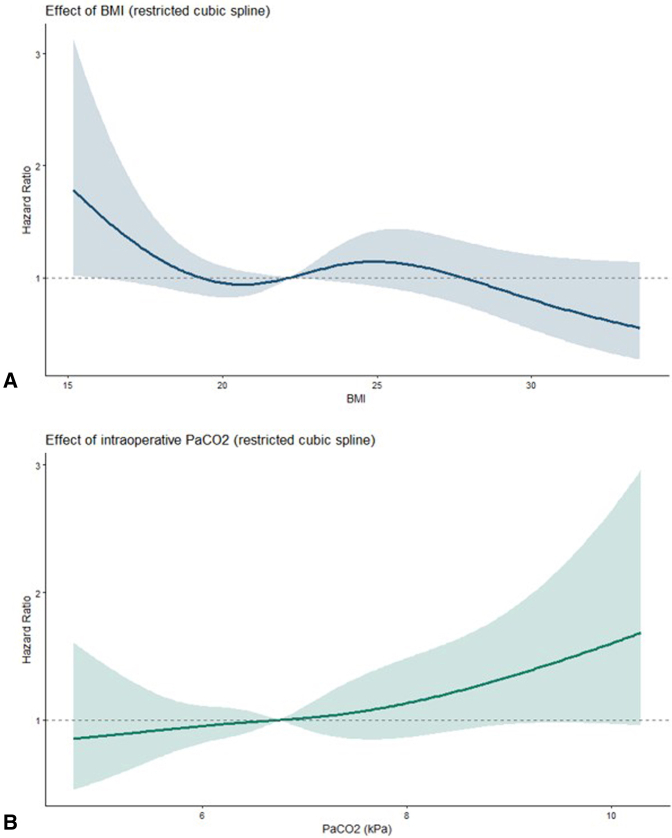
Restricted cubic spline plots for BMI and Paco_2_ at induction of surgery. The figure shows the shape of the hazard functions along the continuous range of both variables, together with 95% CI. A, Nonlinear effect of body mass index (*BMI*) on survival. B, Linear association of Paco_2_ with survival.

To investigate predictors of long-term survival after LVRS, first evaluation of potential nonlinear relationships of key preoperative variables was done. Spline curves showed BMI had an almost linear protective effect, with increased risk in very low BMI ranges (Figure 3, *A*). Paco_2_ at induction of surgery displayed a linear association with mortality, with higher values corresponding to progressively increasing hazard (Figure 3, *B*). Restricted cubic spline analyses showed BMI and Paco_2_ had approximately linear associations with mortality, with no significant nonlinearity (*P* values: BMI 0.85, 0.31; Paco_2_ 0.20, 0.50). Corresponding spline plots are shown in Figure 3; nonlinearity tests are shown in Tables E7 and E8.

### Cohort Effect

**Figure 4:**
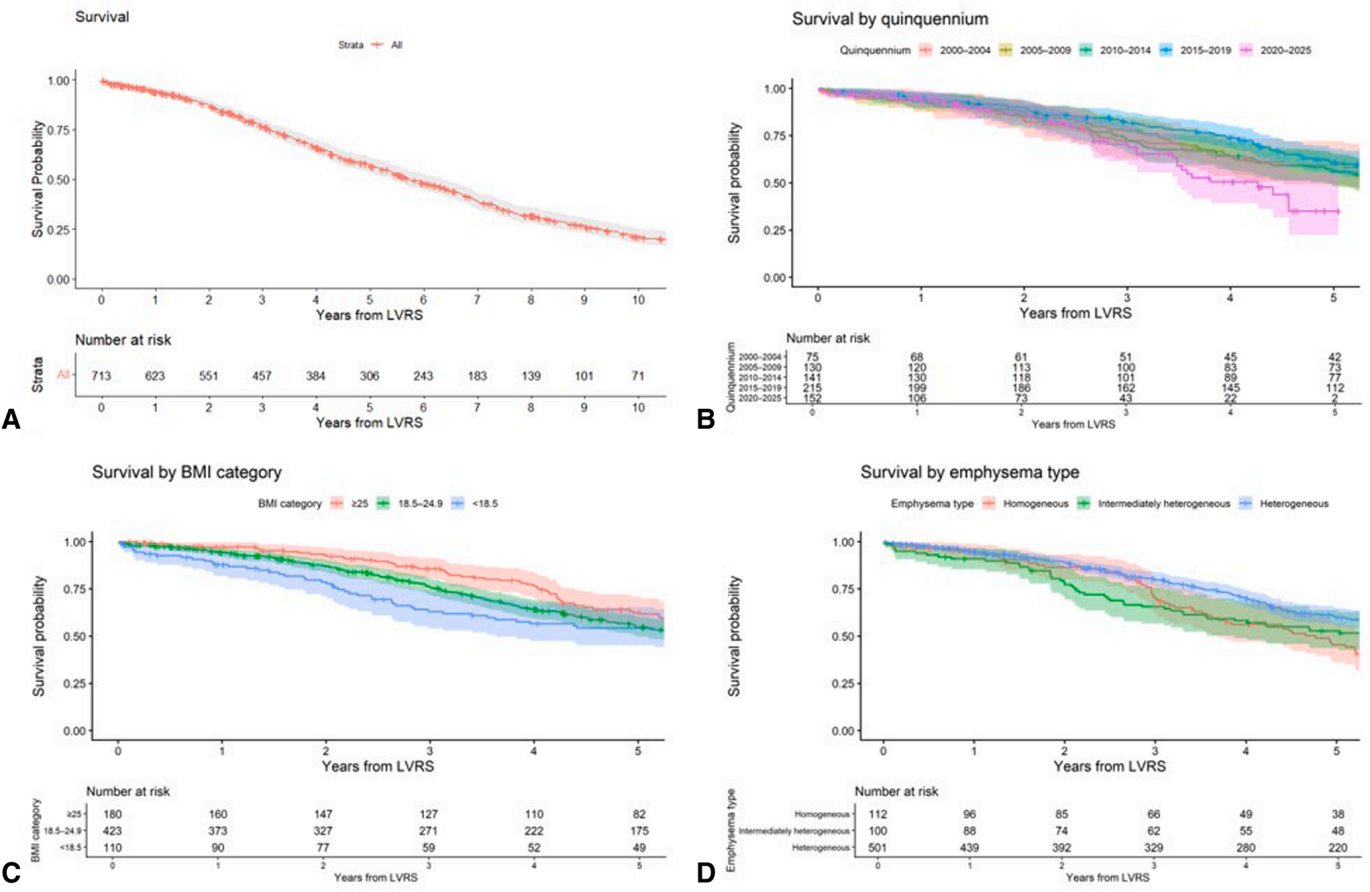
Kaplan-Meier survival curves, with 95% confidence bands. A, Overall survival after lung version reduction surgery (*LVRS*). B, Survival stratified by year (quinquennium) of surgery. C, Survival stratified by body mass index category. D, Survival stratified by emphysema type.

To assess time-related heterogeneity, surgery year was grouped into quinquennia, and Kaplan-Meier analyses were performed for the overall cohort and subgroups (BMI, emphysema morphology, and surgical periods). Survival was highest in normal/elevated BMI and lowest in underweight patients (Figure 4, *C*). Heterogeneous emphysema showed slightly better survival than homogeneous or intermediate forms (Figure 4, *D*). An atypical survival decline occurred in surgeries from 2020 to 2025, consistent with the significant period effect in the multivariable Cox model.

### Determinants of Survival

Survival determinants were evaluated using Cox proportional hazards models for overall survival after LVRS. A multivariable model (Table E7) included BMI, Paco_2_ at induction of surgery, age, COPD classification, morphology, surgical laterality, and complications, with a frailty term for surgery year to account for period heterogeneity. The frailty component was significant (hazard ratio [HR], 1.06; 95% CI, 1.01-1.10; *P* = .0157; Wald *P* = .0078), confirming variability in postoperative mortality.

BMI showed a borderline association with survival (HR, 0.97 per unit increase; 95% CI, 0.94-1.00; *P* = .0767), with no evidence of nonlinearity (*P* = .8112), Paco_2_ at induction of surgery was significantly linked to increased mortality (HR, 1.22; 95% CI, 1.08-1.37; *P* = .0014), also with no significant nonlinear effects (*P* = .1547). Older age showed strongly predicted reduced survival (HR, 1.07 per year; 95% CI, 1.05-1.09; *P* < .001). COPD GOLD classification demonstrated graded but not significant risk increase, although point estimates were large: GOLD II (HR, 4.67; *P* = .1680), GOLD III (HR, 4.66; *P* = .1439), and GOLD IV (HR, 6.61; *P* = .0690) versus GOLD I (overall Wald *P* = .1463). Surgical morphology and laterality were not significantly associated with survival, nor were postoperative complications. Overall, the model strongly predicted survival (global Wald *P* < .0001), with bootstrap validation yielding Harrell's *D* = 0.24 (*C*-index ≈ 0.62).

A second Cox model stratified by morphological confirmed (Table E8) consistent associations for BMI (HR, 0.97; *P* = .070), Paco_2_ (HR, 1.22; *P* = .0012) and age (HR, 1.07; *P* < .0001), with similar GOLD classification patterns, with no statistically significant differences (overall Wald *P* = .1219), but point estimates suggested increasing risk at higher GOLD stages. The frailty term remained significant (HR, 1.06; *P* = .0115), again confirming nonrandom differences across surgical periods. Model discrimination was comparable (Harrell's *D* = 0.22; *C*-index ≈ 0.61). The proportional hazards assumption was verified using Schoenfeld residuals.

## Discussion

Despite COPD's influence on global health and proven benefits of LVRS, the procedure remains underused. We present our experience with what is, to our knowledge, the largest single-center cohort to date. All postoperative functional parameters improved significantly. Mean absolute changes (Δ) at follow-up were: ΔFEV1 +6.9% predicted, ΔTLC −7.5% predicted, ΔRV −762.2 mL, ΔRV/TLC −7.7 percentage points, ΔDLCO +3.2, and Δ6MWD +32 m (all *P* values < .001). Relative to baseline, these correspond to improvements of +22.0% in FEV1, reductions of −14.7% in TLC, −4% in RV, −10.3% in RV/TLC, increases of +9.7% in DLCO, and +2% in walking distance. This effect on lung function was higher to described in other cohorts (ie, change of 22.0% in FEV1 vs 10.5%^14^), even though patients with homogeneous emphysema were also included in our cohort.

Overall, in-hospital mortality was 0.7%, which compared favorably to described rates in the literature ranging from 1.5% to 17.4%.^15^, ^16^, ^17^, ^18^ Also, the 90-day mortality of 2.1% was significantly lower than described in NETT (overall cohort, 7.9%).^2^ This was despite the fact that we included homogenous emphysema and extended our selection criteria for LVRS in a high risk-cohort compared with the NETT: in combination with a well-described target zone(s) and pronounced hyperinflation, we accepted patients with FEV1 <20%, DLCO <20%, or moderate pulmonary hypertension.^19^^,^^20^ An important factor influencing the outcome in patients undergoing LVRS is, in addition to the surgical experience, an extensive experience of the multidisciplinary team dealing with emphysema patients, especially anesthesiology, pulmonology, physiotherapy, and nursing for best perioperative management.^21^ An effective rehabilitation program is paramount to achieve the greatest possible effect of LVRS. Both in-hospital team approach and postoperative rehabilitation are standardized at our center. One-year survival in our cohort was 93.8%, which is significantly higher than described in NETT (LVRS group, 86.5%^2^) and in particular higher than the natural course of the patients with severe emphysema.^17^ Three- and five-year survival rates are influenced by 61 lung transplantations that took place between 19.9 and 60.6 months after LVRS.

The most common perioperative morbidity was PAL in 194 cases (27.2%). This is similar to rates described in literature with 22.9 to 35%.^18^^,^^22^^,^^23^ Our strategy includes the following points: use of buttressed stapling, identification of sufficient resection distance to the fissure, avoidance of resection on the flat surface of the lung to prevent extensive shearing forces while reinflating the lung, and avoidance of surgery in patients with an increased risk of leakage (eg, DLCO <20% in homogeneous emphysema^24^).

The fact that severe pleural adhesions (defined as distributed extensively at the dorsal pleura in at least 2 areas, including the dorsal pleura or the mediastinal pleura) are associated with a higher risk of PAL has been reported previously.^25^ Based on this study, in bilaterally distributed emphysema, we consider termination of surgery at 1 site either if severe adhesions lead to intraoperative air leak or if such adhesions are detected in the overview thoracoscopy at the second side. In our cohort, 54.8% of patients had pleural adhesions.

Low BMI, elevated Paco_2_ at induction of surgery, and older age were independently associated with higher risk of PAL. Underweight, often accompanied by hypoproteinemia, may impair tissue healing and thereby delay closure of air leaks.^26^ Although Paco_2_ has not previously been described as standalone risk factor for PAL, elevated values reflect more advanced COPD or impaired lung function, both of which have been identified as contributors to PAL.^27^ Additionally, older age has been identified as a risk factor for PAL.^28^

The median length of hospital stay was 10 days, which was equal to or slightly longer than that reported in the literature of 6 to 14 days.^23^^,^^29^^,^^30^ As already described in prior publications by our group,^25^ the length of hospital stay is biased by the fact that in our health care system patients have to stay in hospital for direct transfer to inpatient rehabilitation (n = 454). Although more and more patients are discharged directly home with an outpatient rehabilitation program (n = 251), a relevant percentage of patients being referred to rehabilitation centers remain hospitalized longer than medically required. Over the reported period, patients were postoperatively transferred to an intermediate care unit instead of an intensive care unit, which explains the range from 0 to 1 day.

### Predictors of Postoperative Lung Function

Heterogenous emphysema had higher improvement of FEV1 postoperatively. Results from the NETT already identified heterogenous emphysema type as more preferable candidate for LVRS.^3^ In descriptive analyses, bilateral LVRS was associated with slightly larger reductions in RV and RV/TLC compared with unilateral surgery. This effect is simply explained by the greater amount of hyperinflated volume resected in the bilateral approach and has been shown before.^18^

### Predictors for Survival and Complications

Low BMI (<18.5) seems to negatively influence survival in LVRS patients and is associated with a higher rate of complications. This aligns with established evidence that underweight is an independent risk factor for COPD mortality due to increased vulnerability to infections and comorbidities (eg, cardiovascular).^31^^,^^32^ Literature commonly cites BMI <18 as a high-risk threshold for patients undergoing LVRS,^33^ and out cohort suggest BMI <18.5 confers relevant risk, indicating that current cutoffs may require reconsideration. Optimizing nutritional status as part of preoperative enhanced recovery pathways has shown benefits,^30^ and our finding support including postoperative nutrition management as well, particularly because some patients undergoing LVRS may be candidates for lung transplantation.

High Paco_2_ at induction of surgery was associated with a reduced overall survival. Although patients with COPD often tolerate chronically elevated Paco_2_, a marked intraoperative rise may resemble acute exacerbation physiology and could reflect limited ventilatory reserve. Hypercapnia is related to alveolar epithelial dysfunction by impairing resorption of alveolar fluids, suppression of the activation of nuclear factor-kappa and reduction in the innate immunity.^34^^,^^35^ Additionally, hypercapnia has a negative influence on pulmonary circulation (leading to pulmonary hypertension) as well as systemic cardiovascular and musculoskeletal effects by influencing pH. Whether transient hypercapnia during LVRS has similar systemic effects as sustained hypercapnia and therefore leads to reduced survival, remains unclear and warrants further investigations. Preoperative hypercapnia has previously been described as a perioperative risk factor,^36^ and our findings suggest that intraoperative Paco_2_ may additionally support risk stratification. Given its association with poorer outcomes, elevated Paco_2_ could help guide intraoperative decisions, such as limiting the procedure to unilateral LVRS to reduce anesthesia duration. Older age was associated with reduced survival, simply attributed to the natural process.^30^

To explore temporal heterogeneity, the year of surgery was grouped into 5-year intervals, and survival was analyzed by Kaplan-Meier curves across BMI categories, emphysema types, and surgical quinquennia. A transient decline in survival was observed during the 2020 to 2025 period, diverging from the otherwise stable long-term trend. This finding is plausibly related to the COVID-19 pandemic, which may have influenced patient selection, perioperative pathways, and postoperative rehabilitation, as well as long-term outcomes through infection-related morbidity.^37^ Although this cannot be conclusively demonstrated, the pattern aligns temporally with pandemic-related disruptions in elective surgery and rehabilitation services.^38^ As described before, bilateral LVRS was not associated with higher numbers of postoperative complication.^18^

### Limitations

The main limitation pertains to the design of the retrospective, single-center, long-term study, which renders the analysis vulnerable to bias. In the early 2000s, documentation of the postoperative course was poor. Furthermore, the median follow-up was only 36 months because many patients were referred from other institutions or pneumologists. In the Swiss health care system, postoperative follow-ups often occurred externally, and external partners sometimes had a different or no follow-up scheme. Despite efforts to retrieve missing data, long study duration, and external follow-up led to high missingness for several outcomes.

The present study is constrained to LVRS; endobronchial valves were not analyzed and are not listed as a comparison cohort. Lack of quality-of-life data as well as detailed rehabilitation data, which could further reflect the effect of LVRS, is missing. Additional limitations include potential bias from learning curves and device evolution and absence of BMI changes post-LVRS, limiting interpretation of nutritional status. Future data collection will address this.

## Conclusions

LVRS significantly improves all relevant lung function parameters and the 6MWD. In high-volume, experienced centers, these benefits can be achieved with a mortality <1% even in fragile patients, and the present results should therefore be interpreted primarily in this context rather than generalized. All patients with end-stage emphysema should be discussed in an interdisciplinary board and at least be considered for LVRS. Prognostics for survival such as BMI may support perioperative optimization, whereas intraoperative Paco_2_ can assist with risk stratification. Taken together, these findings highlight that rigorous multidisciplinary selection, structured perioperative pathways, and careful attention to nutritional and ventilatory status are as important as the surgical technique itself in determining patient outcomes.

### Webcast

You can watch a Webcast of this AATS meeting presentation by going to: https://www.aats.org/resources/lung-volume-reduction-surgery--9485.

**Figure:**
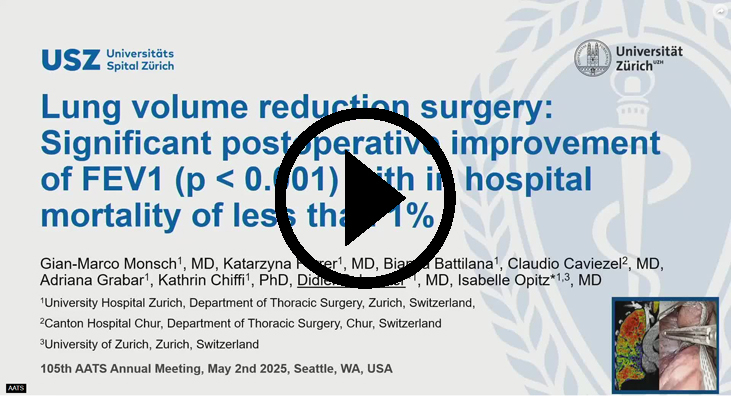

## Conflict of Interest Statement

Dr Gian-Marco Monsch reports relationships with Medtronic (institutional grant) and Intuitive (travel fees). Dr Steinack reports relationships with Intuitive (speaker's fee) and Vertex (speaker's fee). Dr Schneiter reports relationships with Medtronic (institutional grant and advisory board) and Intuitive (proctorship and speaker's fee). Dr Opitz reports relationships with Roche (institutional grant), AstraZeneca (institutional grant, advisory board, and speaker's fee), MSD (advisory board), BMS (advisory board and speaker's fee), Medtronic (institutional grant and advisory board), Intuitive (proctorship and speaker's fee), Sanofi (speaker's fee), Regeneron (advisory board), XVIVO (institutional grant), Siemens (speaker's fee), and Astellas (speaker's fee). All other authors have nothing to disclose.

The *Journal* policy requires editors and reviewers to disclose conflicts of interest and to decline handling or reviewing manuscripts for which they may have a conflict of interest. The editors and reviewers of this article have no conflicts of interest.

## Figures and Tables

**Table E1:** Availability of pre- and posttreatment pulmonary function measurements across outcomes

Variable	N	Pre available	Post available	Pre + Post available	Missing Pre	Missing Post	Both missing
FEV1 (mL)	713	705	451	451	8	262	262
FEV1 (%)	713	705	452	452	8	261	261
RV (mL)	713	670	414	414	43	299	299
RV (%)	713	670	414	414	43	299	299
TLC (mL)	713	675	424	424	38	289	289
TLC (%)	713	675	427	427	38	286	286
RV/TLC	713	684	423	423	29	290	290
DLCO	713	671	394	394	42	319	319
6-min walk distance	713	560	292	292	153	421	421

*FEV1*, Forced expiratory volume in 1 second; *RV*, residual volume; *TLC*, total lung capacity; *RV/TLC*, residual volume to total lung capacity ratio; *DLCO*, diffusing capacity of the lungs for carbon monoxide.

**Table E2:** Baseline demographic and clinical characteristics of patients undergoing lung volume reduction surgery (LVRS), divided by quinquennia (2000-2025)

Patients undergoing LVRS	Overall	2000-2004	2005-2009	2010-2014	2015-2019	2020-2025
No. of cases	713	75	130	141	215	152
Age (y)	65 (59-71)	65 (57-69)	64 (57-69)	66 (60-70)	65 (58-72)	67 (62-73)
Gender						
Male	390 (54.7)	390 (54.7)	39 (52.0)	73 (56.2)	73 (51.8)	123 (57.2)
Female	323 (45.3)	323 (45.3)	36 (48.0)	57 (43.8)	68 (48.2)	92 (42.8)
COPD classification						
I	2 (0.28)	0 (0.0)	1 (0.8)	0 (0.0)	1 (0.5)	0 (0.0)
II	14 (1.96)	0 (0.0)	2 (1.5)	2 (1.4)	5 (2.3)	5 (3.3)
III	198 (27.77)	4 (5.3)	13 (10.0)	33 (23.4)	80 (37.2)	68 (44.7)
IV	399 (55.96)	7 (9.3)	84 (64.6)	104 (73.8)	126 (58.6)	78 (51.3)
Rehabilitation before surgery	70 (9.82)	2 (2.7)	14 (10.8)	13 (9.2)	23 (10.7)	18 (11.8)
Pack-years	45 (40-60)	50 (40-60)	45 (35-62)	48 (35-60)	48 (38-60)	50 (40-60)
Side of emphysema						
Unilateral	171 (23.98)	13 (17.3)	28 (21.5)	31 (22.0)	52 (24.2)	47 (30.9)
Bilateral	542 (76.02)	62 (82.7)	102 (78.5)	110 (78.0)	163 (75.8)	105 (69.1)
Type of emphysema						
Heterogeneous	501 (70.27)	44 (58.7)	75 (57.7)	100 (70.9)	164 (76.3)	118 (77.6)
Homogeneous	112 (15.71)	11 (14.7)	24 (18.5)	20 (14.2)	32 (14.9)	25 (16.5)
Intermediate	100 (14.03)	20 (26.7)	31 (23.9)	21 (14.9)	19 (8.8)	9 (5.9)
Oxygen usage						
Yes	277 (38.85)	25 (33.3)	42 (32.3)	62 (44.0)	78 (36.3)	70 (46.1)
No	436 (61.15)	50 (66.7)	88 (67.7)	79 (56.0)	137 (63.7)	82 (54.0)

Values are presented as median (interquartile range) or n (%) unless otherwise noted. *COPD*, Chronic obstruction pulmonary disease.

**Table E3:** Baseline demographic and clinical characteristics of patients undergoing lung volume reduction surgery (LVRS), divided by type of emphysema

Patients undergoing LVRS	Overall	Heterogeneous	Homogeneous	Intermediately heterogeneous
No. of cases	713	75	130	141
Age (y)	65 (59-71)	65 (59-71)	65 (58-71)	64 (59-69)
Gender				
Male	390 (54.7)	287 (57.3)	56 (50.0)	47 (47.0)
Female	323 (45.3)	214 (42.7)	56 (50.0)	53 (53.0)
COPD classification				
I	2 (0.28)	2 (0.28)	2 (0.40)	0 (0.00)
II	14 (1.96)	14 (1.96)	13 (2.59)	1 (0.89)
III	198 (27.77)	198 (27.77)	150 (29.94)	23 (20.54)
IV	399 (55.96)	399 (55.96)	275 (54.89)	75 (66.96)
Rehabilitation before surgery	70 (9.82)	50 (9.98)	9 (8.04)	11 (11.00)
Pack-years	45 (40-60)	45 (40-60)	50 (40-60)	50 (35-68)
Side of emphysema				
Unilateral	171 (23.98)	125 (24.95)	21 (18.75)	25 (25.00)
Bilateral	542 (76.02)	376 (75.05)	91 (81.25)	75 (75.00)
Oxygen usage				
Yes	277 (38.85)	197 (39.32)	42 (37.50)	38 (38.00)
No	436 (61.15)	304 (60.68)	70 (62.50)	62 (62.00)

Values are presented as median (interquartile range) or n (%) unless otherwise noted. *COPD*, Chronic obstruction pulmonary disease.

**Table E4:** Logistic regression model for complications after lung volume reduction surgery (LVRS)

Covariate (range/categories)	Odds ratio (95% CI)	*P* value	Wald test *P* value
Intercept	14.10 (Coef)	<.0001	–
BMI 19.8-25	1.16 (1.03-1.30)	.0158	.0348 Nonlinear .3100
Paco_2_ 6.11-7.56	0.99 (0.88-1.11)	.8577	.7571 Nonlinear .4558
Age at surgery 59-71 y	0.98 (0.85-1.14)	.8124	.8649
COPD GOLD classification			<.0001
I vs IV	NR	<.0001	
II vs IV	0.98 (0.57-1.67)	<.0001	
III vs IV	3.04 (2.43-3.81)	<.0001	
Morphology			.0007
Homogenous vs heterogenous	0.85 (0.66-1.09)	.2041	
Intermediate vs heterogenous	0.54 (0.38-0.75)	.0009	
LVRS, unilateral vs bilateral	0.70 (0.58-0.84)	.0002	
Total			<.0001 Nonlinear .8274

*Coef*, The intercept is presented as the logistic regression coefficient (β = 14.10) rather than an odds ratio, as exponentiation of the intercept does not yield a clinically meaningful estimate; *BMI*, body mass index; *COPD GOLD*, chronic obstructive pulmonary disease Global Initiative of Chronic Obstructive Lung Disease; *NR*, not reported.

**Table E5:** Logistic regression model for prolonged air leak after lung volume reduction surgery

Covariate (range/categories)	OR (95% CI)	*P* value	Wald test *P* value
Intercept	−15.08 (Coef)	.9798	
BMI 19.70-25.0	0.82 (0.73-0.92)	.018	.0019 Nonlinear .7612
Paco_2_ 6.11-7.56	1.20 (1.08-1.34)	.0009	.0001 Nonlinear .0026
Age at surgery, 59-71 y	1.28 (1.11-1.47)	.006	.0003
COPD GOLD classification			<.0001
I vs IV	NR	<.0001	
II vs IV	1.32 (0.78-2.24)	.27	
III vs IV	0.48 (0.40-0.59)	<.0001	
Morphology			.0001
Homogenous vs heterogenous	1.44 (1.13-1.83)	.004	
Intermediate vs heterogenous	2.19 (1.57-3.05)	.001	
LVRS, unilateral vs bilateral	1.17 (0.98-1.39)	.08	.1092
Total			<.0001 Nonlinear .0529

*Coef*, The intercept is presented as the logistic regression coefficient (β = 14.10) rather than an odds ratio, as exponentiation of the intercept does not yield a clinically meaningful estimate; *BMI*, body mass index; *COPD GOLD*, chronic obstructive pulmonary disease Global Initiative of Chronic Obstructive Lung Disease; *NR*, not reported.

**Table E6:** Multivariable longitudinal analysis (ordinary least squares with cluster-robust SE) of prognostic factors for postoperative lung function after lung volume reduction surgery (LVRS)

Term	FEV1 (mL)	FEV1 (%)	TLC (mL)	TLC (%)	RV (mL)	RV (%)	RV/TLC	DLCO
Months	−83.29 (53.54) [–188.49, 21.92] (3.0-12.0)	−2.88 (0.97) [–4.79, −0.97] (3.0-12.0)	76.72 (177.06) [–271.25, 424.68] (3.0-12.0)	−1.40 (2.49) [–6.30, 3.50] (3.0-12.0)	67.98 (148.95) [–224.74, 360.70] (3.0-12.0)	3.20 (6.24) [–9.07, 15.47] (3.0-12.0)	0.47 (1.26) [–2.01, 2.94] (3.0-12.0)	0.17 (1.16) [–2.11, 2.45] (3.0-12.0)
Age at surgery	−78.35 (47.87) [–172.42, 15.72] (59.0-71.0)	2.28 (0.93) [0.45, 4.11] (59.0-71.0)	−495.54 (193.99) [–876.77, −114.31] (59.0-71.0)	−4.98 (1.79) [–8.49, −1.46] (59.0-71.0)	−149.65 (152.66) [–449.66, 150.37] (59.0-71.0)	−25.45 (4.42) [–34.13, −16.77] (59.0-71.0)	1.82 (0.95) [–0.06, 3.69] (59.0-71.0)	−2.34 (1.36) [–5.01, 0.33] (59.0-71.0)
BMI	45.07 (32.21) [–18.21, 108.35] (19.7-25.0)	−2.02 (1.71) [–5.39, 1.35] (19.7-25.0)	178.27 (151.19) [–118.85, 475.38] (19.7-25.0)	−4.21 (1.65) [–7.45, −0.96] (19.7-25.0)	74.90 (127.73) [–176.13, 325.94] (19.7-25.0)	−2.25 (3.97) [–10.05, 5.55] (19.7-25.0)	−0.63 (0.95) [–2.51, 1.24] (19.7-25.0)	2.54 (1.12) [0.34, 4.73] (19.7-25.0)
PAco_2_ at surgery	−9.36 (36.84) [-81.75, 63.04] (6.1-7.6)	−1.36 (0.95) [–3.24, 0.51] (6.1-7.6)	81.33 (158.87) [–230.89, 393.54] (6.1-7.6)	−3.12 (2.32) [–7.67, 1.43] (6.1-7.6)	99.96 (131.13) [–157.74, 357.67] (6.1-7.6)	0.41 (4.76) [–8.94, 9.76] (6.1-7.6)	0.68 (0.88) [–1.05, 2.41] (6.1-7.6)	−1.61 (1.23) [–4.04, 0.82] (6.1-7.6)
COPD GOLD classification, II vs IV	406.00 (41.82) [323.83, 488.17] (3.0-1.0)	21.23 (3.30) [14.74, 27.72] (3.0-1.0)	−689.70 (253.81) [–1188.50, −190.90] (3.0-1.0)	−0.17 (10.34) [–20.48, 20.15] (3.0-1.0)	−1301.61 (312.52) [–1915.81, −687.42] (3.0-1.0)	−38.48 (15.44) [–68.82, −8.14] (3.0-1.0)	−12.31 (2.68) [–17.58, −7.03] (3.0-1.0)	3.16 (6.15) [–8.93, 15.25] (3.0-1.0)
COPD GOLD classification, III vs IV	146.38 (57.57) [33.25, 259.51] (3.0-2.0)	7.25 (1.43) [4.43, 10.07] (3.0-2.0)	−632.53 (259.63) [–1142.76, −122.30] (3.0-2.0)	−3.35 (2.66) [–8.57, 1.88] (3.0-2.0)	−680.55 (199.81) [–1073.23, −287.88] (3.0-2.0)	−16.17 (6.60) [–29.14, −3.20] (3.0-2.0)	−3.27 (1.43) [–6.09, −0.45] (3.0-2.0)	5.26 (1.92) [1.50, 9.03] (3.0-2.0)
COPD GOLD classification, no classification vs IV	1238.58 (79.64) [1082.10, 1395.07] (3.0-4.0)	40.32 (3.19) [34.06, 46.58] (3.0-4.0)	−476.68 (523.80) [–1506.07, 552.71] (3.0-4.0)	−31.67 (6.12) [–43.69, −19.65] (3.0-4.0)	−1908.23 (488.19) [–2867.66, −948.80] (3.0-4.0)	−100.98 (16.20) [–132.81, −69.15] (3.0-4.0)	−22.88 (3.13) [–29.03, −16.73] (3.0-4.0)	9.14 (2.72) [3.78, 14.49] (3.0-4.0)
Morphology, heterogenous vs homogenous	88.76 (88.19) [–84.53, 262.05] (2.0-1.0)	3.82 (1.63) [0.62, 7.02] (2.0-1.0)	413.25 (270.37) [–118.10, 944.59] (2.0- 1.0)	0.75 (2.84) [–4.84, 6.33] (2.0-1.0)	60.69 (204.31) [–340.83, 462.21] (2.0-1.0)	−3.63 (6.31) [–16.02, 8.76] (2.0-1.0)	−1.95 (1.42) [–4.74, 0.84] (2.0-1.0)	−0.71 (1.73) [–4.11, 2.70] (2.0-1.0)
Morphology, intermediate vs homogenous	−88.90 (75.92) [–238.07, 60.28] (2.0-3.0)	0.02 (1.86) [–3.65, 3.68] (2.0-3.0)	269.72 (508.76) [–730.11, 1269.55] (2.0-3.0)	9.23 (4.94) [–0.48, 18.94] (2.0-3.0)	149.26 (451.60) [–738.25, 1036.78] (2.0-3.0)	17.39 (14.23) [–10.58, 45.35] (2.0-3.0)	1.67 (2.73) [–3.69, 7.03] (2.0-3.0)	−2.87 (2.39) [–7.57, 1.82] (2.0-3.0)
LVRS, unilateral vs bilateral	28.93 (53.79) [–76.76, 134.62] (1.0-2.0)	−0.94 (1.44) [–3.77, 1.90] (1.0-2.0)	202.85 (213.88) [–217.48, 623.18] (1.0-2.0)	0.43 (2.64) [–4.75, 5.62] (1.0-2.0)	163.69 (180.38) [–190.81, 518.19] (1.0-2.0)	1.93 (5.97) [–9.81, 13.66] (1.0-2.0)	0.24 (1.24) [–2.20, 2.68] (1.0-2.0)	−1.53 (1.60) [–4.67, 1.62] (1.0-2.0)
Postoperative complications	−18.91 (52.10) [–121.29, 83.46] (1.0-2.0)	0.48 (1.79) [–3.04, 4.01] (1.0-2.0)	−127.07 (224.72) [–568.70, 314.55] (1.0-2.0)	−3.12 (2.72) [–8.47, 2.23] (1.0-2.0)	128.65 (190.21) [–245.16, 502.45] (1.0-2.0)	4.51 (6.59) [–8.43, 17.46] (1.0-2.0)	2.98 (1.30) [0.42, 5.54] (1.0-2.0)	−0.50 (1.58) [–3.60, 2.59] (1.0-2.0)

Values are expressed as estimated coefficients (SE) [95% CI]. Each cell reports the expected change in postoperative lung function associated with interquartile (Low-High) differences in continuous predictors or with specified category contrasts. Estimates are corrected for within-patient correlation using cluster-robust variance estimation. *FEV1*, Forced expiratory volume in 1 second; *TLC*, total lung capacity; *RV*, residual volume; *BMI*, body mass index; *COPD GOLD*, chronic obstructive pulmonary disease Global Initiative of Chronic Obstructive Lung Disease.

**Table E7:** Cox proportional hazards model with shared frailty term for year of surgery

Covariate (range/categories)	Hazard ratio (95% CI)	*P* value	Wald test *P* value
BMI, restricted cubic spline	0.97 (0.94-1.00)	.0767	.1551 Nonlinear .8112
Paco_2_, restricted cubic spline	1.22 (1.08-1.37)	.0014	.0014 Nonlinear .1547
Age at surgery (y)	1.07 (1.05-1.09)	<.001	<.0001
COPD GOLD classification			.1463
II vs I	4.67 (0.52-41.72)	.1680	
III vs I	4.66 (0.59-36.65)	.1439	
IV vs I	6.61 (0.86-50.53)	.0690	
Morphology			.6397
Homogenous vs heterogenous	1.16 (0.77-1.75)	.4800	
Intermediate vs heterogenous	0.98 (0.66-1.46)	.9265	
LVRS, unilateral vs bilateral	1.18 (0.90-1.56)	.2314	.2194
Postoperative complications, yes vs no	1.21 (0.91-1.62)	.1863	.2262
Year of surgery, frailty term	1.06 (1.01-1.10)	.0157	.0078
Total			<.0001 Nonlinear .3503

Bootstrap validation (10,000 repetitions) showed optimism-corrected discrimination of Harrell's *D* = 0.24, corresponding to a C-index ≈ 0.62, indicating modest but consistent discriminative ability. *BMI*, Body mass index; *COPD GOLD*, chronic obstructive pulmonary disease Global Initiative of Chronic Obstructive Lung Disease; *LVRS*, lung volume reduction surgery; *LVRS*, lung volume reduction surgery.

**Table E8:** Stratified Cox models for overall survival according to emphysema morphology

Covariate (range/categories)	HR (95% CI)	*P*-value	Wald test *P*-value
BMI, restricted cubic spline	0.97 (0.94-1.00)	.070	.1509 Nonlinear .8534
Paco_2_ (restricted cubic spline)	1.22 (1.09-1.37)	.0012	.0018 Nonlinear .1989
Age at surgery (y)	1.07 (1.05-1.09)	<.0001	<.0001
COPD GOLD classification			.1219
II vs I	4.91 (0.54-44.38)	.156	
III vs I	4.76 (0.60-37.88)	.138	
IV vs I	6.91 (0.88-54.44)	.063	
LVRS, unilateral vs bilateral	1.20 (0.91-1.58)	.192	.1806
Postoperative complications, yes vs no	1.17 (0.87-1.57)	.294	.3432
Year of surgery, frailty term	1.06 (1.01-1.10)	.0115	.0071
Total			<.0001 Nonlinear .4280

*BMI*, Body mass index; *COPD GOLD*, chronic obstructive pulmonary disease Global Initiative of Chronic Obstructive Lung Disease; *LVRS*, lung volume reduction surgery.
